# Clinically Actionable Explainable AI in Pulmonary Arterial Hypertension: Endpoints, Calibration, and External Validation. Reply to Pagnoni et al. Toward Clinically Actionable Explainable AI in Pulmonary Arterial Hypertension: Endpoints, Calibration, and External Validation. Comment on “Ledziński et al. Personalized Medicine in Pulmonary Arterial Hypertension: Utilizing Artificial Intelligence for Death Prevention. *J. Clin. Med.* 2025, *14*, 8325”

**DOI:** 10.3390/jcm15051838

**Published:** 2026-02-28

**Authors:** Łukasz Ledziński, Grzegorz Grześk, Michał Ziołkowski, Marcin Waligóra, Marcin Kurzyna, Tatiana Mularek-Kubzdela, Anna Smukowska-Gorynia, Ilona Skoczylas, Łukasz Chrzanowski, Piotr Błaszczak, Miłosz Jaguszewski, Beata Kuśmierczyk-Droszcz, Katarzyna Ptaszyńska, Katarzyna Mizia-Stec, Ewa Malinowska, Małgorzata Peregud-Pogorzelska, Ewa Lewicka, Michał Tomaszewski, Wojciech Jacheć, Michał Florczyk, Ewa Mroczek, Zbigniew Gąsior, Agnieszka Pawlak, Katarzyna Betkier-Lipińska, Piotr Pruszczyk, Olga Dzikowska-Diduch, Katarzyna Widejko, Judyta Winowska-Józwa, Grzegorz Kopeć

**Affiliations:** 1 Department of Cardiology and Clinical Pharmacology, Collegium Medicum in Bydgoszcz, Nicolaus Copernicus University in Toruń, 85-168 Bydgoszcz, Poland; 503345@doktorant.umk.pl (Ł.L.); boldynski@wp.pl (M.Z.); 2 Department of Cardiac and Vascular Diseases, St. John Paul II Hospital in Krakow, 31-202 Krakow, Poland; marcin.waligoora@gmail.com (M.W.); grzegorzkrakow1@gmail.com (G.K.); 3 Pulmonary Circulation Centre, Department of Cardiac and Vascular Diseases, Faculty of Medicine, Jagiellonian University Medical College, 31-202 Krakow, Poland; 4 Center for Innovative Medical Education, Department of Medical Education, Faculty of Medicine, Jagiellonian University Medical College, 30-688 Krakow, Poland; 5 Department of Pulmonary Circulation, Thromboembolic Diseases and Cardiology, Centre of Postgraduate Medical Education, Fryderyk Chopin Hospital in European Health Centre Otwock, 05-400 Otwock, Poland; marcin.kurzyna@ecz-otwock.pl (M.K.); michal.florczyk@ecz-otwock.pl (M.F.); 6 Department of Cardiology, Poznan University of Medical Sciences, 61-701 Poznan, Poland; tatianamularek@wp.pl (T.M.-K.); aniasmuk@wp.pl (A.S.-G.); 7 3rd Department of Cardiology, Faculty of Medical Sciences in Zabrze, Medical University of Silesia, 41-800 Katowice, Poland; iskoczylas@tlen.pl; 8 Cardiology Department, Medical University of Lodz, 91-347 Lodz, Poland; chrzanowski@ptkardio.pl; 9 Department of Cardiology, Cardinal Wyszynski Hospital, 20-718 Lublin, Poland; blaszcz12345@interia.pl; 10 1st Department of Cardiology, Medical University of Gdańsk, 80-210 Gdańsk, Poland; jamilosz@gmail.com; 11 Department of Congenital Heart Disease, Institute of Cardiology, 04-628 Warsaw, Poland; bkusmier@gmail.com; 12 Department of Cardiology, Medical University of Bialystok, 15-276 Bialystok, Poland; kasia.ptaszynska@op.pl; 13 Centre of the European Reference Network for Rare, Low Prevalence or Complex Diseases of the Heart (ERN GUARD Heart), First Department of Cardiology, School of Medicine in Katowice, Medical University of Silesia in Katowice, 40-635 Katowice, Poland; kmiziastec@gmail.com; 14 Pulmonary Department, University of Warmia and Mazury, 10-357 Olsztyn, Poland; ewamalinowska@windowslive.com; 15 Department of Cardiology, Pomeranian Medical University in Szczecin, 70-111 Szczecin, Poland; malgorzata.peregud.pogorzelska@pum.edu.pl; 16 Department of Cardiology and Electrotherapy, Medical University of Gdansk, 80-211 Gdansk, Poland; elew@gumed.edu.pl; 17 Department of Cardiology, Medical University of Lublin, 20-090 Lublin, Poland; tomaszewskimd@gmail.com; 18 2nd Department of Cardiology, Faculty of Medical Sciences in Zabrze, Medical University of Silesia in Katowice, 41-800 Zabrze, Poland; wjachec@interia.pl; 19 Clinic of Heart Diseases, Institute of Heart Diseases, University Clinical Hospital in Wrocław, 50-556 Wrocław, Poland; mroczeke@wp.pl; 20 Department of Cardiology, School of Health Sciences in Katowice, Medical University of Silesia in Katowice, 40-635 Katowice, Poland; zbgasior@gmail.com; 21 Department of Cardiology, National Medical Institute of the Ministry of the Interior and Administration, 02-507 Warszawa, Poland; a.pawlak1@wp.pl; 22 Clinical and Research Department of Applied Physiology, Mossakowski Medical Research Institute, Polish Academy of Sciences, 02-106 Warszawa, Poland; 23 Department of Cardiology and Internal Medicine, Military Institute of Medicine—National Research Institute, 04-141 Warsaw, Poland; kbetkier-lipinska@wim.mil.pl; 24 Department of Internal Medicine and Cardiology with the Center for Diagnosis and Treatment of Venous Thromboembolism, Medical University of Warsaw, 02-005 Warszawa, Poland; piotr.pruszczyk@wum.edu.pl (P.P.); olga.dzikowska-diduch@wum.edu.pl (O.D.-D.); 25 Department of Cardiology, Copper Health Center, 59-300 Lubin, Poland; kwidejko@wp.pl; 26 Department of Cardiology, Provincial Specialist Hospital in Szczecin, 70-111 Szczecin, Poland; j.winowska@gmail.com

## Abstract

The present Reply addresses the commentary by Pagnoni et al. on our recent study exploring explainable artificial intelligence (AI) for mortality risk prediction in pulmonary arterial hypertension (PAH). We acknowledge the importance of several key issues raised by the authors, including endpoint selection, calibration, decision thresholds, and external validation, all of which are central to translating AI-based prognostic models into clinical practice. Our original endpoint, defined as death by the next follow-up visit, was driven by the structure of nationwide registry data and reflects real-world clinical workflows, although we recognize the advantages of predefined time horizons and time-to-event approaches for future analyses. We discuss the trade-off between sensitivity and precision, emphasizing our deliberate prioritization of minimizing false-negative classifications in high-risk patients, while acknowledging the need for structured clinical pathways to manage false-positive results. We further address calibration and threshold selection, underscoring the necessity of additional clinical studies to support intervention-oriented recommendations. The role of phenotypic determinants and modifiable risk factors in enhancing personalization is highlighted as a key direction for future model development. We reaffirm the value of SHAP-based explainability for improving model transparency, while recognizing the need for continued refinement and clinical validation. Finally, we emphasize the strengths and challenges inherent to registry-based analyses, the importance of external validation, and the need for methodologically sound comparisons with established risk calculators. Overall, this exchange underscores the critical role of interdisciplinary collaboration in advancing clinically actionable and interpretable AI solutions for PAH.

## 1. Introduction

The commentary provided by Pagnoni et al. [1] highlights several critical aspects of our study [2], particularly the integration of explainable artificial intelligence (AI) into clinical practice for pulmonary arterial hypertension (PAH). The reviewer’s observations underscore the importance of our core findings, including the challenges associated with endpoint selection, the balance between sensitivity and precision in risk classification, and the interpretability of model outputs. These points align with the broader goal of advancing AI-driven prognostic modeling to enhance clinical decision making.

## 2. Endpoint Selection

We fully agree that the endpoint selection is a critical aspect of prognostic modeling. While our choice of “death by the next follow-up visit” was driven by the structure of the available registry data, we acknowledge that non-uniform follow-up intervals may introduce variability influenced by the healthcare organization rather than intrinsic prognosis. Your suggestion to adopt predefined time horizons or a time-to-event framework is well-taken and aligns with our plans for future studies. Standardizing endpoints will indeed enhance comparability and clinical relevance, and we are actively working toward this goal.

## 3. Sensitivity, Precision, and Clinical Implications

The balance between sensitivity and precision in risk classification is a critical consideration, as highlighted in the commentary. In our study, the decision threshold was optimized to minimize false negatives, prioritizing the reduction in missed high-risk cases. However, we fully recognize the clinical implications of false positives and the need for structured diagnostic and therapeutic pathways to manage these cases effectively. This is an area we aim to explore further, particularly in collaboration with clinical teams to ensure the practical applicability of our model in real-world settings.

## 4. Calibration and Decision Thresholds

The Pagnoni et al. [1] point on calibration and decision thresholds is crucial. While we reported discrimination metrics, we agree that a more comprehensive discussion of calibration and threshold selection would strengthen the clinical translation of our findings. We acknowledge that developing approaches to recommend changes in the context of clinical interventions requires additional clinical studies, and we currently lack such data. This will be a focus in our subsequent analyses, as we aim to provide well-calibrated probabilities and clinically justified thresholds to support decision making in our future research.

## 5. Phenotypic Determinants and Personalization

We appreciate the insights on phenotypic determinants, including sex-specific differences and modifiable factors, which could enhance the personalization of risk predictions. While our current model does not explicitly account for these dimensions, we recognize their importance and are exploring ways to integrate such variables into future iterations of the model. Examples such as the role of vitamin D insufficiency and non-invasive hemodynamic indices provide valuable guidance for expanding the scope of our research.

## 6. SHAP Values and Model Interpretability

The use of SHAP values in our study was intended to enhance the interpretability of the model by quantifying the contribution of each parameter to the predicted risk [3,4]. We agree that this approach holds significant promise for improving the clinical utility of AI-driven predictions. However, we also recognize that further refinement and validation of these methods are necessary, particularly in collaboration with clinical teams, to ensure their practical applicability in diverse clinical settings.

## 7. Registry Data and Analytical Challenges

Pagnoni et al. [1] highlight the unique challenges and opportunities associated with the use of registry data. Unlike clinical trials, which often involve homogeneous study populations due to strict inclusion and exclusion criteria, registry data encompass a broader and more diverse patient population. In our study, the registry included a nationwide cohort of patients treated according to different protocols and diagnosed in various centers [5,6,7]. While this introduces variability in follow-up intervals and therapeutic approaches, it also provides a more realistic representation of clinical practice. We agree that analyzing such datasets is analytically complex but yields conclusions that are more reflective of real-world conditions.

## 8. External Validation and Collaboration

We fully agree with the commentary on the importance of external validation for consolidating the impact of our model. While we currently lack an independent validation dataset, we are actively seeking opportunities to collaborate with other institutions to address this limitation. We also welcome the invitation to collaborate and share data, as joint efforts will undoubtedly accelerate progress in this field.

## 9. Comparison with Existing Risk Calculators

Finally, we acknowledge the importance of comparing our model with existing risk calculators, as suggested in the commentary. This will require careful methodological planning and data standardization, and we appreciate the recognition of the challenges involved in such comparisons. We are committed to undertaking this work in future studies.

## 10. Conclusions

In conclusion, we are grateful for the insightful commentary on our study. The points raised provide valuable guidance for advancing our research and underscore the importance of fostering dialogue and collaboration in the field of AI-driven prognostic modeling. We look forward to further discussions and joint efforts to enhance the integration of AI into clinical practice for PAH.
